# Comparison of Esophageal Dose Parameters in Breast Cancer Radiotherapy With and Without Esophageal Contouring

**DOI:** 10.7759/cureus.113888

**Published:** 2026-08-03

**Authors:** Rajhans Kumar, Pritanjali Singh, Rohit Saini, Jahnavi Kodela

**Affiliations:** 1 Radiation Oncology, All India Institute of Medical Sciences, Patna, Patna, IND; 2 Radiation Oncology, All India Institute of Medical Sciences, New Delhi, New Delhi, IND

**Keywords:** 3dcrt, breast cancer, esophagus delineation, hypofractionation, organ at risk, radiotherapy

## Abstract

Context

This study investigates the effect of esophageal delineation on dose-volume parameters during breast cancer radiotherapy (RT), aiming to optimize treatment planning and reduce the risk of acute esophageal toxicity.

Methods

A retrospective study was conducted on radiotherapy plans from 100 patients with breast cancer (mean age: 45 years) who received three-dimensional conformal radiotherapy (3DCRT) targeting the chest wall and supraclavicular fossa (SCF). All patients were treated with a hypofractionated schedule of 40 Gy in 15 fractions. Initially, esophageal contouring was excluded. In a secondary phase, the esophagus was delineated as an organ at risk (OAR), and the plans were revised accordingly. Dosimetric comparisons included maximum (D_max_), minimum (D_min_), and mean (D_mean_) esophageal doses, along with the volume of the esophagus receiving doses ≥5 Gy (V5), ≥10 Gy (V10), ≥15 Gy (V15), ≥20 Gy (V20), ≥25 Gy (V25), and ≥33 Gy (V33). Additionally, ipsilateral lung volumes (V4, V8, and V16), the Homogeneity Index (HI), and Conformity Index (CI) were assessed to evaluate plan quality.

Results

Delineating the esophagus significantly reduced the radiation dose it received (p < 0.05), without affecting planning target volume (PTV) coverage or compromising plan quality, as indicated by consistent HI and CI values; lung dose parameters remained largely unchanged, although higher mean esophageal doses were observed in left-sided breast cancer cases due to the esophagus’s closer proximity to the supraclavicular fossa, which made beam modulation more challenging.

Conclusions

Incorporating esophageal delineation in 3DCRT planning for breast cancer significantly reduces esophageal dose exposure without compromising dosimetric quality. This adjustment may contribute to minimizing acute esophageal toxicity and enhancing treatment safety.

## Introduction

Breast cancer continues to be the most frequently diagnosed cancer among women worldwide, accounting for approximately 2.3 million new cases and over 670,000 deaths in 2022 [1]. Radiotherapy (RT) remains a cornerstone in the treatment of breast cancer, especially for patients undergoing post-mastectomy therapy that involves irradiation of the chest wall and regional lymphatic regions, including the supraclavicular fossa (SCF) [2]. Despite advancements in RT techniques that have significantly improved locoregional disease control and overall survival, incidental radiation exposure to nearby organs at risk (OARs), notably the heart, lungs, and esophagus, continues to pose challenges, potentially leading to both acute and long-term radiation-induced complications.

Acute radiation esophagitis (ARE) is a clinically significant yet frequently overlooked side effect in breast cancer radiotherapy. Patients may experience symptoms such as odynophagia, dysphagia, and difficulties with nutrition, which can negatively affect both quality of life and adherence to treatment schedules [3-11]. While esophageal toxicity has been well-documented in thoracic malignancies such as lung cancer, its occurrence and implications in breast cancer remain underexplored. This lack of attention is concerning, particularly because of the esophagus’s anatomical proximity to the supraclavicular fossa (SCF) radiation field. The risk is notably higher in left-sided breast cancers, where the esophagus tends to deviate medially in the cervical and upper thoracic regions, placing it closer to high-dose regions during treatment [12]. In lung cancer studies, researchers have identified significant correlations between esophageal dose-volume parameters, particularly the mean dose (D_mean_), and the volumes receiving 10 Gy (V10) and 20 Gy (V20) and the risk of developing radiation-induced esophagitis [8]. Al-Halabi et al. [13] demonstrated that reducing radiation exposure to the contralateral esophagus led to improved dosimetric outcomes and a lower incidence of high-grade esophageal toxicity. Similarly, Ma et al. [12] reported that the use of simultaneous integrated boost intensity-modulated radiotherapy (SIB-IMRT) effectively decreased the mean esophageal dose. Despite these insights, applying such findings directly to breast cancer treatment is limited by key differences in anatomical positioning, radiation field design, and dose fractionation protocols.

Recent efforts, such as the study by Bhaskaran et al. [3], have initiated investigations into esophageal dose exposure during breast cancer radiotherapy, reporting a reduction in mean esophageal dose (D_mean_) through slight rotation (15°-17°) of the supraclavicular (SCF) beam. However, the study was limited by a small patient cohort and did not include comprehensive statistical analysis of volumetric esophageal dose parameters. Similarly, research by West et al. [14] and Yaney et al. [15] proposed dose constraints aimed at reducing the incidence of grade 2 radiation esophagitis, such as maintaining D_mean_ below 11 Gy and V20 under 15%, but these recommendations were derived from conventional fractionation schedules and intensity-modulated radiotherapy (IMRT) plans.

Currently, there is a noticeable gap in the literature regarding the specific impact of esophageal delineation in patients with breast cancer treated with three-dimensional conformal radiotherapy (3DCRT) using hypofractionated protocols, which have become increasingly common in clinical practice. Moreover, very few studies have statistically examined the relationship between esophageal dose-volume parameters and treatment plan quality indicators such as the Conformity Index (CI) and Homogeneity Index (HI), or have assessed how anatomical laterality (left versus right breast) influences dose distribution to the esophagus [8-13].

The purpose of this study was to assess the impact of the dosimetry of esophageal delineation and optimization in the generation of 3DCRT treatment plans for patients with breast cancer undergoing regional nodal irradiation (RNI) using a hypofractionated regimen. The main outcome measure of this study was to determine the reduction of esophageal dose parameters such as mean esophageal dose (D_mean_) and dose-volume parameters, whereas the secondary objectives included the evaluation of PTV coverage, Conformity Index (CI), Homogeneity Index (HI), dose parameters of the lungs, and the correlation of esophageal dose parameters and quality of treatment plans. It was predicted that the combination of esophageal delineation with optimization of supraclavicular fields would help in reducing the dose to the esophagus without compromising the quality of treatment plans. However, since this study is dosimetry-based, any observed reduction in the dose to the esophagus and associated radiation esophagitis is to be considered only as hypothesis-generating and requires validation in future clinical trials.

This study aims to fill existing gaps in the literature by evaluating treatment plans from 100 patients with breast cancer (50 with left-sided and 50 with right-sided disease), comparing scenarios with and without esophageal delineation using three-dimensional conformal radiotherapy (3DCRT). All patients received hypofractionated radiotherapy with a total dose of 40 Gy delivered in 15 fractions. Unlike prior studies, the supraclavicular (SCF) beam angles were adjusted by 15°-20° to help reduce esophageal dose exposure. Notably, this is the first study in this context to apply Pearson correlation analysis to explore associations between esophageal dose-volume parameters and plan quality metrics, including the Conformity Index (CI) and Homogeneity Index (HI). By combining practical treatment adaptations with robust statistical evaluation, this study offers new evidence supporting the inclusion of esophageal contouring in routine breast radiotherapy planning to enhance safety without compromising treatment quality.

## Materials and methods

Data acquisition

This study analyzed data from 100 patients with breast cancer treated between January 2021 and April 2024 at the Department of Radiation Oncology, All India Institute of Medical Sciences (AIIMS), Patna, India. The study was approved by the Institutional Ethics Committee of All India Institute of Medical Sciences (AIIMS), Patna (IEC approval number: AIIMS/Pat/IEC/1380, approval date: December 30, 2024). The study population consisted of an equal number of patients with right-sided (n = 50) and left-sided (n = 50) breast cancer. The mean esophageal volume was 21.06 ± 6.75 cm³ in right-sided cases and 19.48 ± 6.76 cm³ in left-sided cases.

All included patients had undergone mastectomy followed by adjuvant radiotherapy targeting the chest wall and supraclavicular fossa (SCF). Patients were excluded if they had significant thyroid gland enlargement or if their radiotherapy was confined to the breast tissue without SCF irradiation.

Simulation and treatment planning

Patients were positioned supine on a breast board with both arms elevated above the head and the head turned to the contralateral side. CT simulation was performed using a 16-slice GE Healthcare CT simulator with 2 mm slice thickness. A uniform 7-mm margin was applied to expand the clinical target volume (CTV) to the planning target volume (PTV). Treatment planning was performed using 6 MV photon beams for patients with thin chest walls and 10 MV photon beams for patients with larger chest wall separations. The chest wall was irradiated using two opposed tangential photon fields, whereas the supraclavicular fossa (SCF) was treated using a single anterior photon field. A single-isocenter half-beam block technique with asymmetric jaws was employed for field matching between the tangential and SCF fields. The field-in-field (FiF) technique was used, when necessary, to improve dose homogeneity and reduce dose hotspots. A 5-mm tissue-equivalent bolus was applied in all treatment plans.

Retrospective contouring was done for the esophagus according to the Radiation Therapy Oncology Group (RTOG) contouring guidelines. Contouring was done from the cricoid cartilage level down to the inferior border of the supraclavicular fossa (SCF) field in the planning CT scans using the Monaco Treatment Planning System (TPS) version 6.0.0.13. The same contouring criteria were used for all the patients to maintain consistency during comparison of dose distribution. In addition, equivalent dose in 2 Gy fractions (EQD2) values for esophageal D_max_ and D_mean_ were calculated using the linear-quadratic model with an α/β ratio of 3 Gy, which is the commonly accepted value for late-responding normal tissues, including the esophagus.

**Figure 1:**
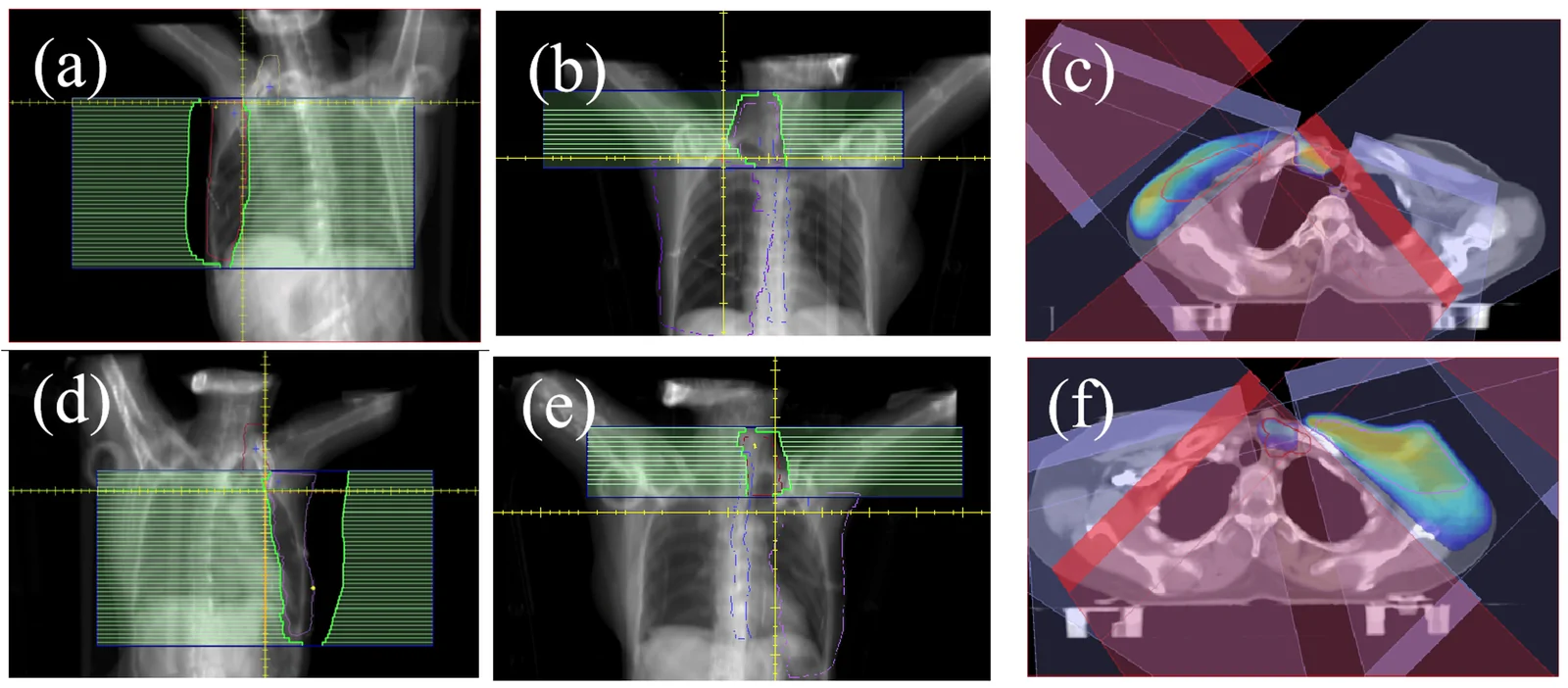
Representative 3DCRT field arrangement illustrating the half-beam block field matching technique between the CW and SCF fields (a) Right CW BEV, (b) right SCF BEV, (c) composite right CW-SCF plan showing the 95%-107% isodose distribution, (d) left CW BEV, (e) left SCF BEV, and (f) composite left CW-SCF plan showing the 95%-107% isodose distribution, demonstrating accurate field matching and dose homogeneity at the junction 3DCRT: three-dimensional conformal radiotherapy, CW: chest wall, SCF: supraclavicular fossa, BEV: beam eye view

**Figure 2:**
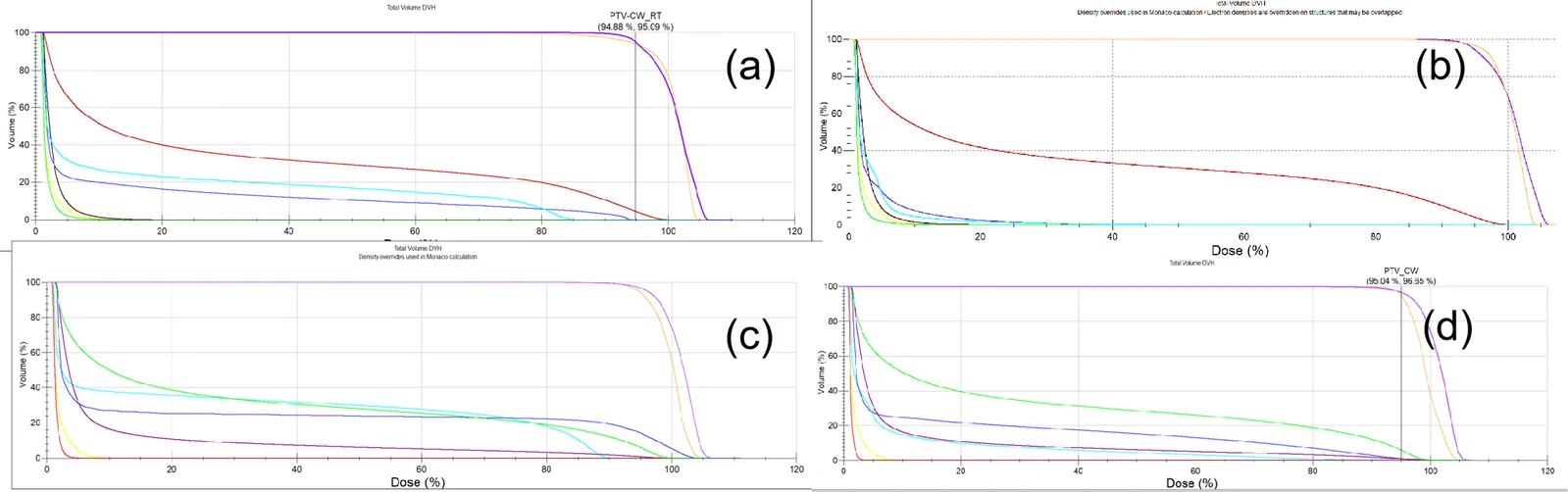
DVH comparison of treatment plans with and without esophageal optimization in right- and left-sided breast cancer (a) Right breast without esophageal optimization, (b) right breast with esophageal optimization, (c) left breast without esophageal optimization, and (D) left breast with esophageal optimization Structures are color-coded as follows: PTV-CW (purple), PTV-SCF (gold), contralateral breast (yellow), spinal cord (cyan), esophagus (blue), heart (royal blue), right lung (red), and left lung (green). DVH: dose-volume histogram, PTV-CW: planning target volume, CW: chest wall, SCF: supraclavicular fossa

In phase I (Group A), the treatment plans (Figures 1, 2) were retrospectively planned without the delineation of the esophagus. In phase II (Group B), the esophagus was retrospectively delineated from the cricoid cartilage level up to the inferior border of the supraclavicular fossa (SCF) field. Two 3D conformal radiotherapy (3DCRT) plans were retrospectively planned for each patient using the same planning CT scans: one for Group A without the delineation of the esophagus and the other for Group B with the esophagus delineation and SCF beam-angle optimization. Both plans delivered a dose of 40 Gy in 15 fractions. The chest wall irradiation was administered through two opposite tangential photon beams, whereas the SCF irradiation was performed using only one anterior photon beam. A single-isocenter half-beam block with asymmetric jaws and 0° collimator angle technique was employed to ensure optimal field matching and uniformity of junction doses. For the left-side case, the angles of gantries for the medial and lateral tangential beams were set to 300°-310° and 120°-130°, respectively; for the right-side case, the angles were 50°-60° and 230°-240°, respectively. To avoid the esophageal overdose without compromising PTV coverage, the individualization of the gantry angle for the SCF (by approximately 15°-20°) was performed for the right-side case (340°-345° for the mirror left-side case). The FiF technique was used when required for achieving better homogeneity of PTV doses. Other parameters of planning for all pairs of cases, including beam energy, field arrangement, prescription dose, optimization goals, and dose calculation algorithm, remained unchanged.

For Group B, beam angles for the SCF field were adjusted typically within a gantry range of 15°-20° to reduce esophageal exposure while preserving target coverage. Both patients underwent treatment with two different planning techniques based on the same CT data set, the same target volume definition, beam energy, beam arrangement, prescribed dose, and optimization criteria. The sole difference in the planning technique between Group A and Group B was that for Group B, the esophagus was defined with the SCF beam angles adjusted to decrease esophageal dose.

A plan was deemed clinically acceptable if 95% of the PTV received 95% of the prescription dose (V95% ≥ 95%) and if both the HI and CI were maintained at acceptable levels. Dose hotspots inside the PTV were restricted to no more than 107% of the prescription dose, while doses to the OARs, especially the esophagus and the ipsilateral lung, remained within acceptable limits according to institutional planning criteria and RTOG guidelines.

Dosimetric evaluation included maximum (D_max_), minimum (D_min_), and mean (D_mean_) esophageal doses, along with the volume of esophagus receiving ≥5 Gy (V5), ≥10 Gy (V10), ≥15 Gy (V15), etc. Additional parameters analyzed were ipsilateral lung volumes receiving ≥4 Gy (V4), ≥8 Gy (V8), and ≥16 Gy (V16), as well as plan quality metrics such as the Homogeneity Index (HI) and Conformity Index (CI).

Comparative statistical analyses were performed between Group A and Group B, as well as between left- and right-sided breast cancer cases. Pearson correlation coefficients were calculated to explore the relationship between esophageal dose parameters and overall plan quality indices.

Dosimetric evaluation and comparison

Dosimetric parameters were meticulously extracted from the dose-volume histogram (DVH) for comprehensive evaluation. Key metrics such as the maximum dose (D_max_), minimum dose (D_min_), and mean dose (D_mean_) to the esophagus were recorded. Since patients were treated with a hypofractionated regimen of 2.67 Gy per fraction rather than the conventional 2 Gy per fraction, the biologically equivalent dose in 2 Gy fractions (EQD2) was calculated for both D_max_ and D_mean_. This conversion allowed for meaningful comparison with previously published data [3].

Additionally, the percentage of esophageal volume receiving doses at or above specific thresholds ≥5 Gy (V5), ≥10 Gy (V10), ≥15 Gy (V15), ≥20 Gy (V20), ≥25 Gy (V25), and ≥33 Gy (V33) was carefully recorded. To evaluate the overall quality of the treatment plans, key volumetric parameters were analyzed, with particular emphasis on the Homogeneity Index (HI) and the Conformity Index (CI). Treatment planning was carried out using the Monaco Treatment Planning System (TPS) version 6.0.0.13. Contouring of the clinical target volume (CTV), planning target volume (PTV), and organs at risk (OARs) followed the Radiation Therapy Oncology Group (RTOG) and standard atlas-based guidelines: \[ CI = \frac{V_{PTV}}{V_{TV}} \]

The HI measures the uniformity of dose distribution within the planning target volume (PTV), reflecting how evenly the prescribed radiation dose is delivered throughout the target.

HI is calculated using the following equation [16,17]:\[ HI = \frac{D_{2\%} - D_{98\%}}{D_{\mathrm{prescription}}} \]

D2​% is the dose received by 2% of the PTV, D98​% is the dose received by 98% of the PTV, and Dprescription is the prescribed dose. A lower HI indicates better dose homogeneity.

The CI evaluates how well the high-dose region conforms to the shape of the PTV [16,17]. It is given by the following equation [16,17]:\[ CI = \frac{V_{PTV}}{V_{TV}} \]

VPTV is the volume of the PTV receiving the prescription dose, and VTV is the total volume enclosed by the prescription isodose (prescription isodose volume). A higher CI indicates that the radiation is more confined to the PTV, minimizing exposure to surrounding healthy tissues. Given the significance of limiting the dose to the ipsilateral lung in radiotherapy for Ca breast, attention was directed toward the percentage volume of the lung on the same side of the PTV receiving doses ≥4 Gy (V4), ≥8 Gy (V8), and ≥16 Gy (V16), which was selected accordingly to the RTOG 1005. These parameters were meticulously derived from the DVH for comprehensive evaluation and comparison.

Statistical analysis

Statistical analysis was conducted using Jamovi (third-generation software), an open-source platform widely used for its robust and user-friendly statistical tools. For visualization and plotting of comparative dosimetric data, OriginPro software was utilized, given its high-resolution graphing and analytical capabilities. All statistical tests were two-sided, and a p-value < 0.05 was considered statistically significant.

Additionally, Pearson correlation coefficients were calculated to assess the strength and direction of linear relationships between selected dosimetric variables and treatment planning metrics. Correlation values near +1 indicate a strong positive linear relationship, while those near -1 suggest a strong negative linear association. Accompanying p-values determined the statistical significance of these correlations.

To enhance the clarity of results, bar plots and comparative charts were generated to visually illustrate differences between the two groups (with and without esophagus delineation), enabling more intuitive interpretation of key trends. Furthermore, the Pearson correlation coefficient was calculated using the comparison of paired parameters of dosimetry in both Group A (without the esophagus demarcated) and Group B (with the esophagus demarcated). The correlation tests have been conducted separately on both right- and left-sided samples. Since it is an exploratory study and is not designed to test multiple hypotheses, there is no correction for multiple comparisons.

## Results

**Figure 3:**
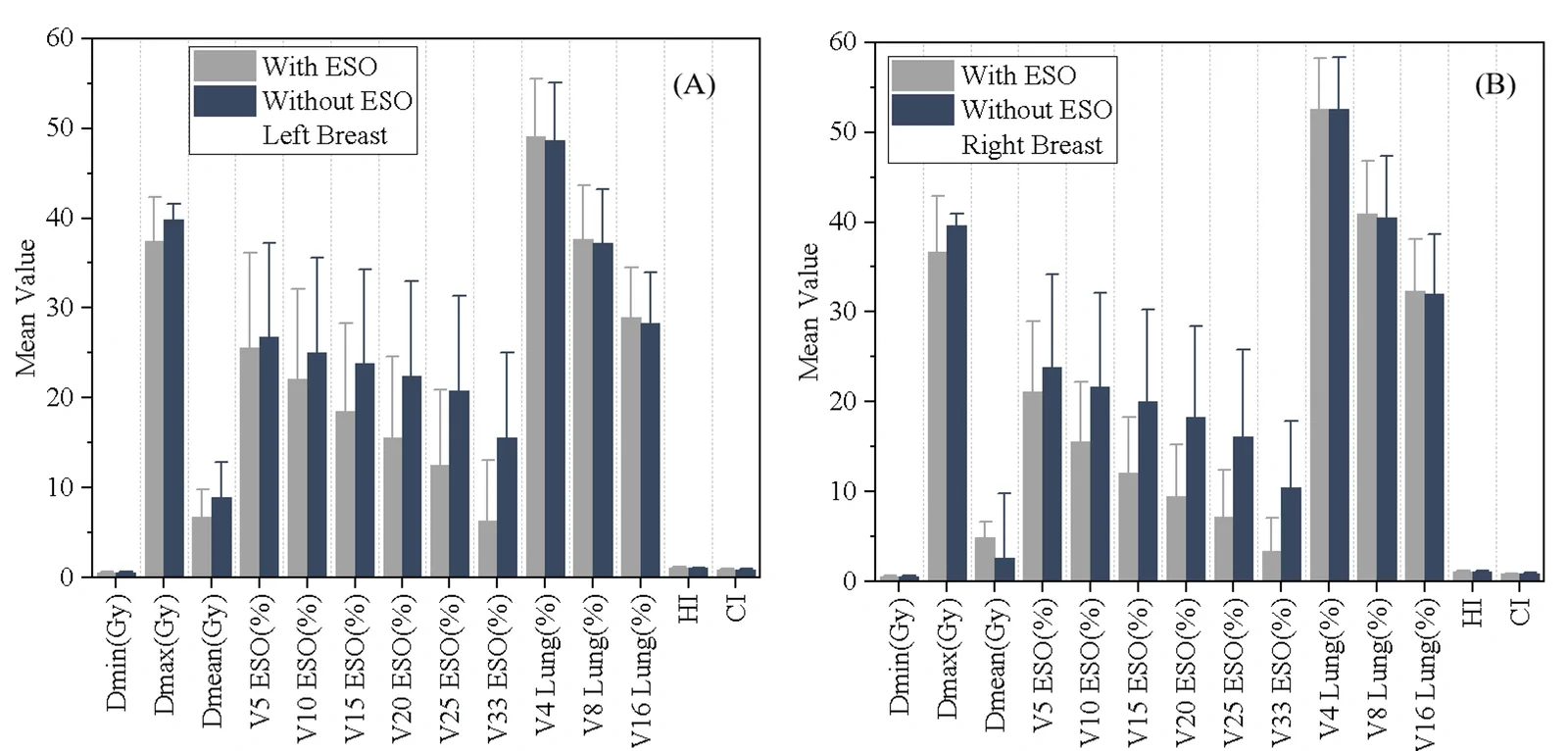
Dosimetric comparison of esophageal dose parameters in breast radiotherapy plans with and without ESO optimization: (A) left-sided breast plans and (B) right-sided breast plans ESO: esophageal, D_min_: minimum dose, D_max_: maximum dose, D_mean_: mean dose, Vn es: volume received by n(number) Gy in esophagus, HI: Homogeneity Index, CI: Conformity Index

**Figure 4:**
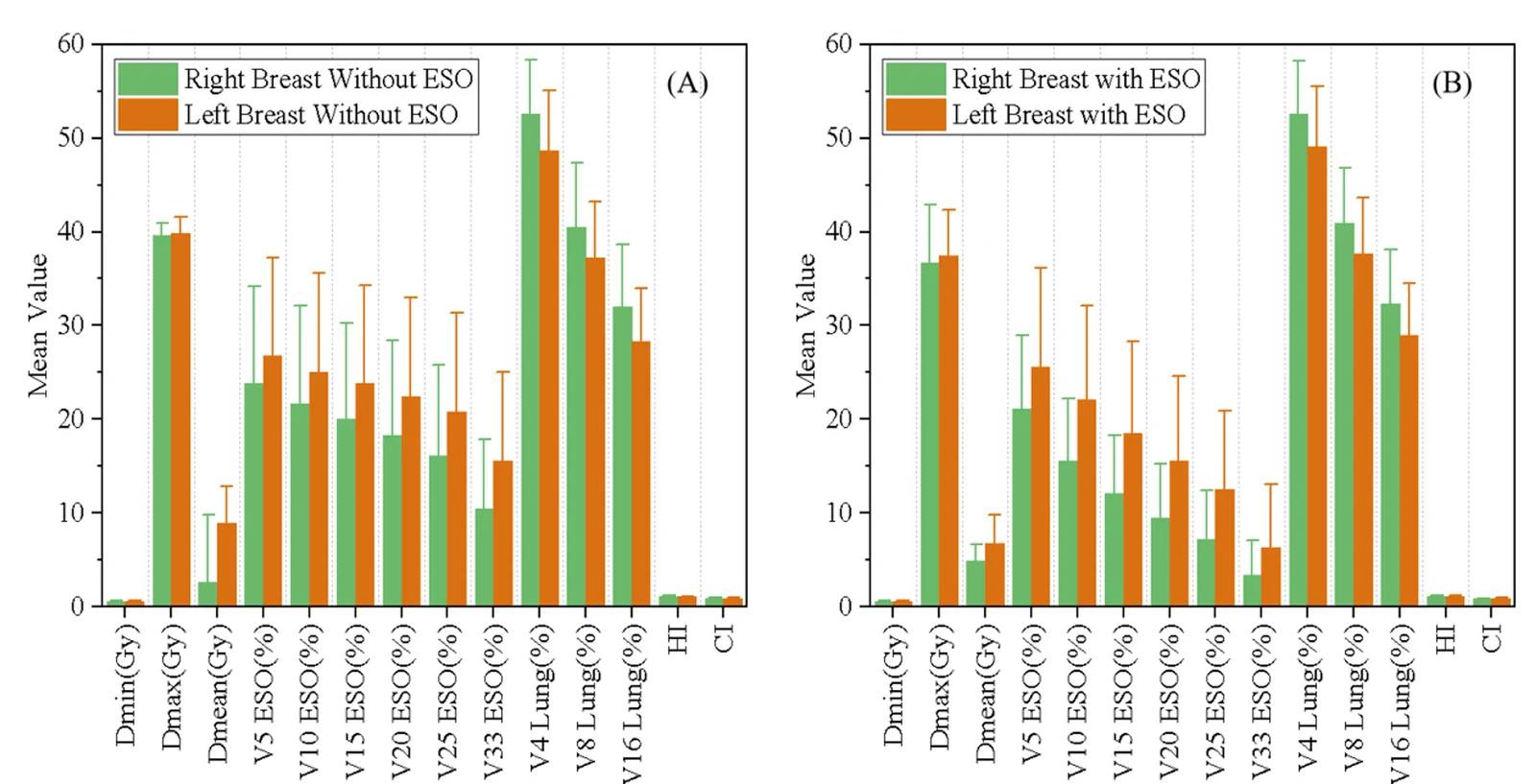
Dosimetric comparison of esophageal dose parameters between right-sided and left-sided breast radiotherapy plans: (A) without ESO optimization (Group A) and (B) with ESO (Group B) ESO: esophageal, D_min_: minimum dose, D_max_: maximum dose, D_mean_: mean dose, Vn es: volume received by n(number) Gy in esophagus, HI: Homogeneity Index, CI: Conformity Index

The study included 100 patients equally divided between right-sided (n = 50) and left-sided (n = 50) breast cancer. For each patient, two retrospective treatment plans were generated: Group A (without esophageal delineation) and Group B (with esophageal delineation). Dosimetric parameters for the planning target volume (PTV) and organs at risk (OARs) were extracted from dose-volume histograms (DVHs) and compared. The results are summarized in Tables 1-3 and Figures 3, 4.

**Table 1:** Comparison of dosimetric parameters between Group A and Group B in right-sided versus left-sided plans D_min_: minimum dose, D_max_: maximum dose, D_mean_: mean dose, Vn es: volume received by n(number) Gy in esophagus, HI: Homogeneity Index, CI: Conformity Index, SD: standard deviation, eso: esophagus

	Group A					Group B				
	Right breast (mean ± SD)	Left breast (mean ± SD)	Mean difference	p-value	Pearson’s r	Right breast (mean ± SD)	Left breast (mean ± SD)	Mean difference	p-value	Pearson’s r
D_min_ (Gy)	0.55 ± 0.11	0.54 ± 0.13	0.01	0.981	-0.004	0.53 ± 0.10	0.55 ± 0.13	-0.01	0.335	0.144
D_max_ (Gy)	39.66 ± 1.30	39.86 ± 1.69	-0.19	0.493	-0.102	36.67 ± 6.23	37.43 ± 4.88	-0.75	0.984	-0.003
D_mean_ (Gy)	7.20 ± 2.65	8.89 ± 3.90	-6.23	0.953	0.009	4.92 ± 1.79	6.80 ± 2.98	-1.87	0.641	0.070
V5 eso (%)	23.87 ± 10.29	26.81 ± 10.36	-2.94	0.575	-0.084	21.09 ± 7.87	25.55 ± 10.61	-4.46	0.605	-0.077
V10 eso (%)	21.70 ± 10.44	25.04 ± 10.52	-3.34	0.687	-0060	15.57 ± 6.60	22.13 ± 9.97	-6.56	0.826	-0.033
V15 eso (%)	20.02 ± 10.28	23.83 ± 10.46	-3.81	0.923	-0.014	12.08 ± 6.25	18.54 ± 9.73	-6.46	0.792	0.039
V20 eso (%)	18.32 ± 10.09	22.48 ± 10.53	-4.16	0.846	0.029	9.48 ± 5.83	15.6 ± 9.02	-6.12	0.831	0.032
V25 eso (%)	16.14 ± 9.67	20.83 ± 10.49	-4.69	0.667	0064	7.23 ± 5.23	12.54 ± 8.33	-5.31	0.961	0.007
V33 eso (%)	10.51 ± 7.40	15.56 ± 9.45	-5.05	0.32	0.148	3.37 ± 3.69	6.38 ± 6.67	-3.01	0.693	-0.059
V4 lung (%)	52.54 ± 5.77	48.66 ± 6.46	3.88	0.383	0.13	52.60 ± 5.66	49.1 ± 6.42	3.5	0.530	0.094
V8 lung (%)	40.48 ± 6.83	37.2 ± 6.0	3.28	0.104	0.24	40.87 ± 5.89	37.64 ± 6.02	3.23	0.787	-0.041
V16 lung (%)	32.05 ± 6.56	28.3 ± 5.7	3.75	0.095	0.247	32.38 ± 5.76	28.99 ± 5.52	3.39	0.959	0.008
HI	1.14 ± 0.04	1.12 ± 0.03	0.02	0.385	0.134	1.15 ± 0.04	1.13 ± 0.05	0.02	0.270	0.164
CI	0.93 ± 0.03	0.93 ± 0.03	0	0.928	-0.014	0.92 ± 0.03	0.93 ± 0.03	-0.01	0.934	0.012

**Table 2:** Comparison of dosimetric parameters between Group A (without esophageal delineation) and Group B (esophagus delineation) in left-sided plans D_min_: minimum dose, D_max_: maximum dose, D_mean_: mean dose, Vn es: volume received by n(number) Gy in esophagus, HI: Homogeneity Index, CI: Conformity Index, SD: standard deviation, eso: esophagus

	Group A (mean ± SD)	Group B (mean ± SD)	Mean difference	p-value	Pearson’s r
D_min_ (Gy)	0.54 ± 0.13	0.55 ± 0.13	0.01	<0.001	0.568
D_max_ (Gy)	39.86 ± 1.69	37.43 ± 4.8	-2.43	0.197	0.194
D_mean_ (Gy)	8.89 ± 3.93	6.80 ± 2.98	-2.09	<0.001	0.455
V5 eso (%)	26.81 ± 10.36	25.55 ± 10.61	-1.26	<0.001	0.653
V10 eso (%)	25.04 ± 10.52	22.13 ± 9.97	-2.91	<0.001	0.672
V15 eso (%)	23.83 ± 10.46	18.54 ± 9.73	-5.29	<0.001	0.616
V20 eso (%)	22.48 ± 10.53	15.6 ± 9.02	-6.88	<0.001	0.606
V25 eso (%)	20.83 ± 10.49	12.54 ± 8.33	-8.29	<0.001	0.576
V33 eso (%)	15.56 ± 9.45	6.38 ± 6.67	-9.18	<0.001	0.524
V4 lung (%)	48.66 ± 6.46	49.1 ± 6.42	0.44	0.009	0.378
V8 lung (%)	37.2 ± 6.01	37.64 ± 6.02	0.44	0.002	0.432
V16 lung (%)	28.3 ± 5.7	28.99 ± 5.52	0.69	<0.001	0.547
HI	1.12 ± 0.03	1.13 ± 0.05	0.01	0.424	0.119
CI	0.93 ± 0.03	0.93 ± 0.03	0	<0.001	0.684

**Table 3:** Comparison of dosimetric parameters between Group A (without esophageal delineation) and Group B (esophagus delineation) in right-sided plans D_min_: minimum dose, D_max_: maximum dose, D_mean_: mean dose, Vn es: volume received by n(number) Gy in esophagus, HI: Homogeneity Index, CI: Conformity Index, SD: standard deviation, eso: esophagus

	Group A (mean ± SD)	Group B (mean ± SD)	Mean difference	p-value	Pearson’s r
D_min_ (Gy)	0.55 ± 0.11	0.53 ± 0.10	0.0131	<0.001	0.958
D_max_ (Gy)	39.66 ± 1.30	36.67 ± 6.23	2.9872	0.576	0.082
D_mean_ (Gy)	7.2 ± 2.6	4.92 ± 1.79	-2.2766	0.237	0.174
V5 eso (%)	23.87 ± 10.29	21.09 ± 7.87	2.78	<0.001	0.798
V10 eso (%)	21.70 ± 10.44	15.57 ± 6.60	6.13	<0.001	0.482
V15 eso (%)	20.02 ± 10.28	12.08 ± 6.25	7.94	0.064	0.267
V20 eso (%)	18.32 ± 10.09	9.48 ± 5.83	8.84	0.306	0.149
V25 eso (%)	16.14 ± 9.67	7.23 ± 5.23	8.91	0.669	0.063
V33 eso (%)	10.51 ± 7.40	3.37 ± 3.69	7.14	0.259	0.164
V4 lung (%)	52.54 ± 5.77	52.60 ± 5.66	-0.06	<0.001	0.859
V8 lung (%)	40.48 ± 6.83	40.87 ± 5.89	-0.39	<0.001	0.794
V16 lung (%)	32.05 ± 6.56	32.38 ± 5.76	-0.33	<0.001	0.792
HI	1.140 ± 0.042	1.15 ± 0.04	-0.01	<0.001	0.512
CI	0.93 ± 0.03	0.92 ± 0.03	0.01	<0.001	0.6

Right breast analysis

In the right breast cohort (Table 1 and Figure 3B), for radiotherapy plans between Group A and Group B, key dosimetric parameters were evaluated to assess differences in radiation exposure to the esophagus, lungs, and overall plan quality.

The esophageal dose was consistently higher in Group A across most metrics. The D_min_ showed a statistically significant difference favoring Group B, with Group A receiving slightly higher doses. While the D_max_ and D_mean_ doses were also greater in Group A, these differences were not statistically significant. Volume-based parameters revealed that a significantly larger percentage of the esophagus received low doses (V5 and V10) in Group A compared to Group B (p < 0.001), indicating better esophageal sparing in Group B. Although higher dose volumes (V15-V33) were also lower in Group B, these did not reach statistical significance, although the trend was consistent.

Lung dose parameters, including V4, V8, and V16, were almost identical between the two groups. Although the differences were statistically significant, the absolute magnitude of these differences was small. This suggests that both planning approaches provided comparable lung protection.

With respect to plan quality, the Homogeneity Index (HI) and Conformity Index (CI) were slightly better in Group A. Although these differences reached statistical significance, the values were very close between the groups and well within acceptable clinical limits, indicating that both groups maintained good plan quality. These results demonstrate that Group B offers a clear dosimetric advantage in reducing esophageal radiation dose, especially at lower dose levels, while preserving lung sparing and overall plan quality. These findings demonstrate improved esophageal dosimetric sparing with Group B while maintaining comparable lung dose parameters and overall plan quality.

Left breast analysis

In the left-sided cohort (Table 2 and Figure 3A), where the esophagus lies closer to the supraclavicular region, more pronounced dosimetric differences were observed between Group A and Group B.

This study presents a detailed dosimetric comparison between Group A and Group B in left-sided breast radiotherapy, focusing on critical organs at risk, including the esophagus and ipsilateral lung, as well as overall treatment plan quality metrics.

The esophageal dose D_min_ was comparable between the two groups, with Group B demonstrating a marginal but statistically significant increase (0.55 ± 0.13 Gy versus 0.54 ± 0.13 Gy; p < 0.001). Although the esophageal dose D_max_ was higher in Group A (39.86 ± 1.69 Gy) compared to Group B (37.43 ± 4.89 Gy), this difference did not reach statistical significance (p = 0.197). Importantly, the esophageal dose D_mean_ was significantly elevated in Group A relative to Group B (8.89 ± 3.93 Gy versus 6.81 ± 2.99 Gy; p < 0.001), indicating greater esophageal dose reduction with the Group B planning approach.

Analysis of esophageal dose-volume metrics further underscored this finding; Group B exhibited statistically significant reductions across all evaluated thresholds (V5 through V33). Notably, the volume of esophagus receiving ≥33 Gy was markedly lower in Group B (6.38% ± 6.67) compared to Group A (15.56% ± 9.45), indicative of enhanced protection of this critical structure.

Regarding ipsilateral lung exposure, Group B demonstrated slightly increased low-dose volumes (V4, V8, and V16) relative to Group A, with all differences reaching statistical significance (p < 0.01). However, the absolute magnitude of these differences was minimal, suggesting that both techniques afford comparable lung sparing in clinical terms.

Evaluation of plan quality metrics revealed no significant difference in Homogeneity Index (HI) between groups (p = 0.424), whereas Conformity Index (CI) values were equivalent but statistically favored Group B (p < 0.001), reflecting marginally improved target conformity.

These data indicate that Group B offers a clear dosimetric advantage in reducing esophageal dose at all clinically relevant thresholds without compromising lung protection or overall plan quality in left-sided breast radiotherapy. These findings demonstrate improved esophageal dosimetric sparing with Group B without compromising lung dose parameters or treatment plan quality.

Right breast versus left breast analysis

In Table 3 and Figure 4A, 4B (both Group A and Group B), left-sided breast radiotherapy plans showed slightly higher esophageal dose parameters (D_mean_ and V5-V33) compared to right-sided plans, although none of these differences were statistically significant. For example, esophageal D_mean_ increased from 7.20 ± 2.65 Gy (right) to 8.89 ± 3.93 Gy (left) in Group A, and from 4.92 ± 1.79 Gy to 6.80 ± 2.98 Gy in Group B. Lung dose parameters (V4, V8, and V16) were marginally higher in right-sided plans across both groups, while Homogeneity Index and Conformity Index remained similar between right-sided and left-sided treatments.

Overall, laterality showed minimal impact on dosimetric parameters, with no significant differences observed in either group.

Correlation analysis and dosimetric insights

Pearson correlation analysis demonstrated positive associations between the mean esophageal dose (D_mean_) and intermediate- to high-dose esophageal volume parameters (V20-V25), with correlation coefficients of approximately r ≈ 0.8 (p < 0.01) (Table 1). These findings indicate that treatment plans associated with higher mean esophageal doses also tended to have larger esophageal volumes receiving intermediate and high radiation doses. As this analysis was exploratory, the observed correlations should be interpreted as measures of association rather than evidence of clinical benefit or causality. This finding indicates that treatment plans delivering a higher average dose to the esophagus are also associated with greater volumes of the esophagus receiving doses in the intermediate to high range, indicating an association between increasing mean esophageal dose and higher intermediate- to high-dose esophageal volumes.

## Discussion

With growing emphasis on minimizing radiation-induced toxicity, particularly to organs at risk (OARs), optimizing breast cancer radiotherapy plans has become essential. Although extensive literature addresses lung and heart sparing, limited studies focus on esophageal dosimetry, especially in patients with breast cancer receiving regional nodal irradiation (RNI). This study critically contributes to this gap by analyzing the impact of esophagus-sparing techniques on dosimetric outcomes.

Esophagus dose reduction along with consistency and divergence with lung cancer literature

Our findings highlight the potential of three-dimensional conformal radiotherapy (3DCRT), when combined with strategic beam-angle optimization, to substantially reduce esophageal dose. As shown in Table 1, the esophageal dose D_mean_ in Group B for right-sided breast plans was significantly lower than in Group A (4.92 ± 1.79 Gy versus 7.20 ± 2.65 Gy). A similar trend was observed in left-sided plans (Table 2), where Group B also demonstrated a lower D_mean_ compared to Group A (6.80 ± 2.98 Gy versus 8.89 ± 3.93 Gy). Notably, these values are consistently lower than those reported by Bhaskaran et al. [3], who found D_mean_ values of 8.36 Gy (right) and 13.86 Gy (left) for esophagus-sparing plans, further supporting the efficacy of optimized 3DCRT techniques in minimizing esophageal exposure.

This comparison demonstrates the feasibility and effectiveness of our planning approach in reducing the esophageal dose while maintaining adequate PTV coverage in reducing esophageal dose through simple SCF beam rotation (15°-20°), without compromising PTV coverage. In the study by Bhaskaran et al., SCF rotation was limited to 15°-17°. Differences in planning technique, patient anatomy, and treatment planning methodology may have contributed to the observed differences in dosimetric outcomes.

Our results also align with findings from thoracic oncology. Ma et al. [12] demonstrated the effectiveness of integrated-boost IMRT in reducing esophageal D_mean_ in lung cancer. Although our study employed 3DCRT rather than IMRT, the consistent reduction in D_mean_ illustrates that careful planning and beam optimization can yield similar protective benefits in different anatomical contexts.

Al-Halabi et al. [13] showed that sparing the contralateral esophagus reduced grade 3 toxicities in lung cancer. While we did not assess clinical toxicity in this study, there was a significant reduction in V5 and V10, which have been associated with radiation esophagitis in previous studies.

Esophagus and lung dose trade-off and its clinical relevance and implications

Our data also confirm the anatomical complexity of left-sided breast cancer planning. The esophagus tends to curve leftward near the supraclavicular region, making it more difficult to spare in left-sided plans. Table 3 supports this: the D_mean_ was consistently higher in left-sided cases, regardless of the group. This is in line with the study by Yaney et al. [16], who emphasized the need for tight dosimetric constraints D_mean_< 11 Gy, V10 < 30%, and V20 < 15% to reduce esophagitis risk. In our study, Group B largely met these thresholds, reinforcing the effectiveness of esophagus-sparing approaches even in anatomically challenging scenarios.

However, in contrast to studies using IMRT (West et al. [14]), which reported higher D_mean_ and a non-significant trend toward more frequent grade 2 radiation esophagitis (G2RE), our 3DCRT-based plans achieved lower D_mean_ values with better dose homogeneity (HI difference < 0.01 between groups; p > 0.05). These findings suggest that careful beam optimization using 3DCRT can achieve favorable esophageal dosimetric outcomes while maintaining acceptable target coverage and plan quality. The observed reductions in D_mean_ and low-dose volume parameters (V5-V20) demonstrate improved dosimetric sparing of the esophagus. Whether these dosimetric improvements translate into clinical benefits requires prospective studies incorporating toxicity outcomes. By lowering D_mean_ and V10, both critical thresholds for predicting esophagitis treatment plans can be made safer without compromising target coverage or homogeneity.

It is important to note that the reduction in esophageal dose did not compromise treatment quality. In addition to the significant decrease in D_mean_ and intermediate- to high-dose esophageal volume parameters, the optimized plans maintained comparable PTV coverage, Conformity Index, Homogeneity Index, and overall plan quality. These findings indicate that esophageal delineation and optimization represent a promising strategy for improving organ at risk sparing while preserving treatment quality. However, prospective clinical studies are required to determine whether these dosimetric improvements translate into reduced radiation-induced esophageal toxicity.

Additionally, our findings help contextualize esophageal sparing within breast cancer treatment protocols, where regional irradiation is becoming increasingly standardized. The present findings support consideration of esophageal delineation during treatment planning, particularly for left-sided regional nodal irradiation. However, multicenter studies incorporating clinical outcome data are required before routine clinical implementation can be recommended.

Limitations of the study

Despite the proven benefits of esophagus-sparing 3DCRT planning in patients with breast cancer undergoing regional nodal irradiation, there are several limitations that should be addressed. First, the analysis was mainly dosimetric and requires further assessment in terms of its impact on clinical endpoints, such as acute and late esophagitis. Second, the study was limited to a single center and was conducted on patients who received optimized 3DCRT treatment; thus, the results need to be replicated through multicenter research and comparison with other advanced techniques, such as IMRT, volumetric modulated arc therapy (VMAT), and proton therapy. Despite these limitations, the findings demonstrate that esophageal delineation during 3DCRT planning can improve esophageal dosimetric sparing without compromising target coverage or overall treatment plan quality. The Pearson correlation analysis demonstrated positive associations between mean esophageal dose and intermediate- to high-dose esophageal volume parameters. These findings indicate that treatment plans with higher mean esophageal doses also tended to irradiate larger esophageal volumes at clinically relevant dose levels.

## Conclusions

The results of this research show that optimization of esophageal delineation and planning in the case of hypofractionated 3D-conformal radiotherapy reduces the mean dose to the esophagus and other dose-volume parameters associated with the esophagus without changing PTV coverage and treatment quality in comparison with the case where esophageal contouring is not applied. This evidence proves the necessity of esophageal delineation during the process of breast radiotherapy planning in order to increase the sparing of this organ at risk. However, since this research was focused only on dosimetric analysis, it does not provide any information on changes in the rate of esophageal toxicity.
